# Supplementary material for: Chromothripsis during telomere crisis is independent of NHEJ, and consistent with a replicative origin
Source: Genome Res. 2019 May;29(5):737–49. doi: 10.1101/gr.240705.118 (PMC6499312; doi:10.1101/gr.240705.118)
Supplement: Supplemental Material [file supp_gr.240705.118_Supplemental_file_1.zip › contigs/annotated_contigs/DB56/contig.2.DB56_length_358_mean_cov_2.18156424581.docx]

**DB56_length_358_mean_cov_2.18156424581**

```
TGAGTGAGAATATATGGTGTTTGGTTTTTTGTTCTTGCGATAGTTTACTGAGAATGATGATTTCCAATTTCATCCATGACCCTACAAAG
 >chr9:105552876-105553179 + E=2e-165 p=1e-03
GACATGAACTCATCATTTTTTATGGCTGTGTAGTATTCCATGGTGTATATGTGCCACATTTTCTTGATCCAGTCTAGCATTGTTGGACA

TTTGGGTTGGTTCCAAGTCTTTGCTATTGTGAATAATGCCGCAGTAAACATACATGTGCATGTGTCTTTATAGCAGCATGATTTATATT

CCTTTGGGTATATACCCAGTAATGGGATGGCT|GGGT|ATATACCCAAAGGAATATAAATCATGCTGCTATAAAGACACATGCACATGT
                                 >chr1:203581425-203581484 - E=5e-21
ATGT
```
